# Imeglimin for Type 2 Diabetes Mellitus: Its Efficacy and Insight Into the Potential Benefit for Renal and Liver Function

**DOI:** 10.7759/cureus.66322

**Published:** 2024-08-06

**Authors:** Asuka Uto, Yuki Ishinoda, Takamasa Asaga, Yuki Tanahashi, Ai Kobayashi, Hitomi Meshino, Maki Okazaki, Kengo Tomita, Akira Kasuga, Naoki Oshima

**Affiliations:** 1 Department of Endocrinology and Metabolism, National Defense Medical College, Tokorozawa, JPN; 2 Department of Gastroenterology and Hepatology, National Defense Medical College, Tokorozawa, JPN; 3 Department of Nephrology, National Defense Medical College, Tokorozawa, JPN

**Keywords:** transient elastography (fibroscan), microalbuminuria (ma), nonalcoholic fatty liver disease (nafld), diabetic nephropathy (dn), imeglimin

## Abstract

Introduction

Imeglimin is a novel oral antihyperglycaemic drug used to treat type 2 diabetes mellitus (T2DM). In 2022, its clinical use was approved in Japan; however, there is limited data on its practical efficacy. Thus, we retrospectively investigated the clinical efficacy of imeglimin for six months at the National Defense Medical College, Tokorozawa, Japan.

Material and methods

We conducted a single-center retrospective analysis to elucidate the efficacy of imeglimin in the treatment of T2DM. Ten patients were enrolled, and their biomarkers and geographic data were analyzed. The primary endpoint was the change in HbA1c level at six months after imeglimin treatment compared to the baseline values. Other demographic and laboratory parameters, including sex, age, BMI, renal function, liver function, lipid profile, and transient elastography data, were also analyzed.

Results

A significant improvement in the HbA1c levels (8.1 % at baseline to 6.9 % at six months after treatment, P value = 0.01) was observed in this study, suggesting that imeglimin is a promising option for treating T2DM. In addition, no negative effects on renal function were observed, and albumin levels tended to decrease from baseline values. Among the nonalcoholic fatty liver disease (NAFLD) cases, liver conditions, especially fat content, tended to improve in this short-term period.

Conclusions

Imeglimin is suggested to have a beneficial effect not only on glycemic control but also on renal and liver function. However, further studies are required to better understand the long-term efficacy of this drug.

## Introduction

Diabetes mellitus (DM) is defined as chronic hyperglycemia with insufficient insulin function; its microvascular and macrovascular complications can cause life-threatening events [1,2]. While type 1 DM is caused by an absolute deficiency of insulin due to the autoimmune destruction of β cells and is categorized as an endocrine disorder, type 2 DM (T2DM) is mainly caused by insulin resistance, which is accompanied by an intemperate lifestyle such as overeating and lack of exercise and is now categorized as a non-communicable disease, which is defined by the World Health Organization (WHO) as a current global health issue [3].

Diabetic nephropathy (DN), a major complication of DM, manifests as proteinuria and progressive decline in renal function, which eventually leads to end-stage renal disease (ESRD) and requires renal replacement therapy. DN is the primary cause of hemodialysis, and its economic cost has gradually increased [4]. DN typically develops sequentially from the incipient stage of microalbuminuria to ESRD. Early intervention for DN is important to slow or even prevent the progression of this process. Large clinical trials have revealed that the most useful drugs for DN management are angiotensin-converting-enzyme inhibitors [5], angiotensin 2 blockers [6], sodium-glucose cotransporter 2 inhibitors [7], and mineralocorticoid receptor antagonists [8]. However, the current management of DN is far from adequate, and new therapeutic options are coveted.

T2DM is often comorbid with other metabolic diseases, such as dyslipidemia and hypertension, and these comorbidities increase the morbidity and mortality of vascular diseases. Among these, non-alcoholic fatty liver disease (NAFLD) is an underestimated but important comorbidity of T2DM. NAFLD is a condition in which excessive accumulation of fat is detected in the liver without heavy alcohol intake (< 20 g/day), ranging from simple steatosis to nonalcoholic steatohepatitis [9]. The pathogenesis of NAFLD is initiated by insulin resistance, and T2DM is a favorable condition for NAFLD development. A recent study revealed that patients with NAFLD are susceptible to cardiovascular diseases [10]. Therefore, similar to DN, early interventional treatments for NAFLD are necessary to prevent the development of subsequent critical conditions; however, there are no applicable NAFLD-specific medical agents.

Imeglimin is a first-in-class drug, which is classified into the “glimin” group, a tetrahydro triazine-containing class of oral antidiabetic agents. Although the precise mechanism remains unclear, imeglimin is presumed to act on mitochondria and has dual actions: driving insulin secretion from the pancreas and improving insulin signaling in the liver and muscles [11]. In the pancreas, the action of imeglimin is thought to protect mitochondrial respiratory chain complexes by reducing reactive oxygen species and consequently promoting insulin secretion. Its action on the liver and muscles is thought to improve insulin sensitivity. The safety and efficacy of imeglimin were examined in the TIMES study [12-14], the clinical trial of imeglimin. The effectiveness of this drug in monotherapy or combination therapy with insulin or other hypoglycemic agents has been confirmed in these trials.

Japan was the first country to apply imeglimin in a clinical setting. Imeglimin was approved by the Japanese Pharmaceuticals and Medical Devices Agency for the treatment of T2DM in June 2021 [15]. Considering its unique pharmacological mechanism, this drug has great potential for the treatment of T2DM, but there is little real-world data on its efficacy, and further information is desired to better understand the selection of patients for whom this drug is suitable. We conducted a retrospective study to evaluate the　effectiveness of imeglimin in patients who attended the National Defense Medical College Hospital for the treatment of T2DM. The main aim of this study was to evaluate the effectiveness of imeglimin for glycemic control. We found that initiation of imeglimin improves not only diabetic control but also has a beneficial effect on DN and NAFLD conditions. Imeglimin may be a novel drug for the treatment of T2DM accompanied by DN and NAFLD.

## Materials and methods

Study design and participants

This single-center retrospective study was conducted to verify the efficacy of imeglimin. The study included patients with T2DM who were prescribed imeglimin for six months between February 2022 and December 2022. Imeglimin was administered for glycemic control at the physician’s discretion. There were no other changes or additions to the baseline treatment in this study period. The primary outcome was the change in glycated hemoglobin (HbA1c) levels six months after the initiation of imeglimin. Baseline demographic data of the participants, such as age, sex, body mass index (BMI), waist circumference, concomitant antidiabetic drugs, and adverse events, were obtained from medical records. BMI was calculated as weight in kg divided by squared height in meters (kg/m^2^). We also obtained the following clinical laboratory data: complete blood count, HbA1c, blood glucose, C-peptide, aspartate aminotransferase (AST), alanine aminotransferase (ALT), γ-glutamyl transpeptidase (γGT), low-density lipoprotein cholesterol (LDL-C), high-density lipoprotein cholesterol (HDL-C), triglycerides, creatinine (Cr), estimated glomerular filtration rate (eGFR), and urinary albumin-to-creatinine ratio (UACR). C-peptide index (CPI) was calculated as the ratio of serum C-peptide to blood glucose levels.

This study was approved by the Ethics Committee of the National Defense Medical College and conducted in accordance with the Declaration of Helsinki.

Definition of DN stages

We defined DN stages as follows: (1) normoalbuminuria, UACR level of less than 30 mg/g; (2) microalbuminuria, any UACR level between 30 and 300 mg/g; and (3) macroalbuminuria, UACR level of more than 300 mg/g.

Diagnosis of NAFLD

None of the patients had etiologies of hepatitis virus infection, heavy alcohol intake (daily ethanol consumption of < 30 g in men and < 20 g in women), or drug-induced liver injury. The fatty liver index (FLI) was used for NAFLD diagnosis [16]. FLI was calculated based on the following equation; FLI = [e^(0.953 × ln(triglycerides) + 0.139 × BMI + 0.718 × ln(γGT) + 0.053 × waist circumference - 15.745)^]/[1 + e^(0.953 × ln(triglycerides) + 0.139 × BMI + 0.718 × ln(γGT) + 0.053 × waist circumference - 15.745)^] × 100. FLI ≥ 60 was used to diagnose NAFLD.

FibroScan examination

Those who were diagnosed with NAFLD were examined the liver stiffness and steatosis by FibroScan (Echosens, Paris, France) [17]. Experienced operators conducted the examination. After an overnight fast, patients were instructed to lay in the supine position with the right arm elevated. Estimations were made using a transducer probe starting in the right upper quadrant at the level of the right liver lobe. The quantitative values of liver stiffness and steatosis were evaluated as liver stiffness measurement (LSM) and controlled attenuation parameter (CAP), respectively. Up to three estimations were performed on every patient with LSM and CAP outcomes. LSM was presented in decibels per meter (dB/m) and CAP was expressed as kilopascal (kPa).

Statistical analysis

Statistical analyses were performed using the JMP version 17 software (SAS Institute Japan, Tokyo, Japan). All data are expressed as mean ± standard error (SE) or as medians and interquartile range as appropriate. Within-group comparisons of changes from baseline were performed using the paired t-test or Wilcoxon signed-rank test. Statistical significance was set at P < 0.05.

## Results

Baseline characteristics of the study group

Ten patients were enrolled in this study. The demographic and baseline characteristics are shown in Table 1.

**Table 1:** Baseline characteristics of enrolled patients. Data are presented as numbers (%) for categorical variables and as means (± standard error of the mean, SEM). BMI, body mass index; HbA1c, glycated hemoglobin; OHA, oral hypoglycemic agent; DPP-4, dipeptidyl peptidase-4; SGLT2, sodium-glucose cotransporter 2; GLP-1, glucagon-like peptide-1.

Variables	n = 10
Age (year)	63.1 ± 3.2
Men/Women (n)	9/1
BMI (kg/m^2^)	28.8 ± 1.6
Waist circumference (cm)	97.1 ± 4.3
HbA1c at baseline (%)	8.4 ± 0.4
BMI ≧ 25 kg/m^2^, n (%)	9 (90)
Medication usage	
Insulin, n (%)	4 (20)
OHA, n (%)	8 (80)
Biguanide, n (%)	8 (80)
Sulfonylurea, n (%)	2 (20)
DPP-4 inhibitor, n (%)	3 (30)
SGLT2 inhibitor, n (%)	5 (50)
GLP-1 receptor agonist, n (%)	3 (30)

The mean age was 63.1 years old, and the baseline HbA1c level was 8.4 %. The average BMI of the participants showed the existence of obesity (28.8 kg/m^2^), and their waist circumferences were 97.1 cm, which was supposed to represent visceral fat > 100 cm^2^. Only one woman was included in this study.

Two patients were administered imeglimin monotherapy. The remaining eight patients all received metformin. Of the 10 patients, five received dipeptidyl peptidase 4, and four received SGLT2 inhibitor therapy. Insulin was administered to four patients.

Effect of imeglimin on glycemic control

Six months after the initiation of imeglimin treatment, HbA1c levels improved significantly compared to baseline values (Table 2). CPI at six months was elevated as compared to that before the initiation of imeglimin, suggesting the promotion of insulin secretion from the pancreas (P = 0.14). BMI significantly decreased in the months period (28.8 kg/m^2^ at baseline to 27.8 kg/m^2^ at six months, P = 0.03). Waist circumference showed a subtle decline from baseline levels.

**Table 2:** Changes in parameters compared between baseline and six months after initiation of imeglimin. Data are presented as means (± standard error of the mean, SEM). P values are calculated by paired t-test (vs. baseline parameters). CPI, C-peptide index; LDL-C, low-density lipoprotein cholesterol; HDL-C, high-density lipoprotein cholesterol; AST, aspartate aminotransferase; ALT, alanine aminotransferase; γGT, γ-glutamyl transpeptidase; Cr, creatinine; eGFR, estimated glomerular filtration rate; N/A, not available.

	Baseline	Six months	Reference range	P
HbA1c (%)	8.4 ± 0.4	6.8 ± 0.2	4.6 - 6.2	0.01
Fasting glucose (mg/dL)	142 ± 6.4	124 ± 7.8	65 - 110	0.09
C peptide (ng/mL)	2.2 ± 0.4	2.4 ± 0.4	0.6 - 2.1	0.71
CPI	1.6 ± 0.3	2.1 ± 0.4	N/A	0.14
LDL-C (mg/dL)	110 ± 11	94 ± 11	< 140	0.31
HDL-C (mg/dL)	51 ± 4.2	49.3 ± 3.0	42 - 62	0.77
Triglycerides (mg/dL)	163 ± 24	152 ± 21	30 - 150	0.74
AST (IU/L)	22 ± 3.5	20 ± 1.3	8 - 30	0.55
ALT (IU/L)	27 ± 5.4	22 ± 3.0	5 - 35	0.50
γGT (IU/L)	35 ± 8.9	26 ± 5.2	7 - 70	0.40
Cr (mg/dL)	0.99 ± 0.04	0.98 ± 0.04	0.61 - 1.13	0.98
eGFR (mL/min/1.73 m^2^)	59.5 ± 2.6	59.4 ± 2.4	≥ 60	0.98
BMI (kg/m^2^)	28.8 ± 1.6	27.8 ± 1.6	N/A	0.03
Waist circumference (cm)	97.1 ± 4.3	96.7 ± 4.3	N/A	0.95

Effect of imeglimin on renal function

**Figure 1:**
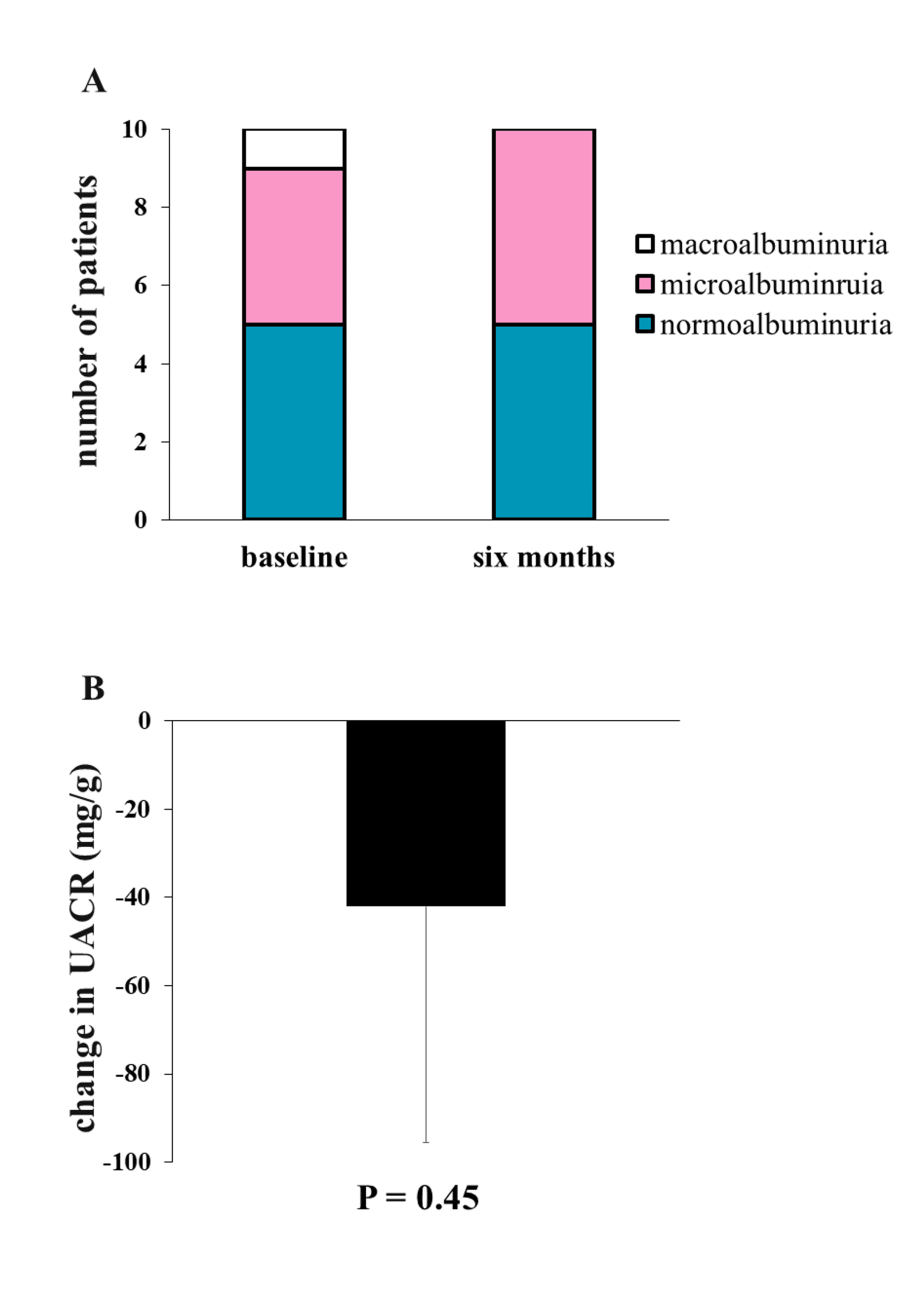
Changes in DN stages and UACR levels from baseline to six months after imeglimin. A: The changes in DN stages of enrolled patients. B: The change in UACR at six months after imeglimin treatment. Data are presented as means (± standard error of the mean, SEM). P values are calculated by a paired t-test (vs. baseline parameters). DN, diabetic nephropathy; UACR, urinary albumin-to-creatinine ratio

We assessed the safety of imeglimin on renal function by evaluating the eGFR transition. At six months of its initiation, the eGFR values remained unchanged from the baseline, suggesting the potential safety of imeglimin on renal function (59.5 mL/min/1.73 m^2^ to 59.4 mL/min/1.73 m^2^, P = 0.98) (Table 2). We also investigated whether the initiation of imeglimin treatment would have any effect on DN by assessing the changes in UACR. At baseline, four patients showed normoalbuminuria, and five patients had microalbuminuria. One patient developed macroalbuminuria, suggesting advanced renal damage (Figure 1A). The change in the DN stage after the initiation of imeglimin is shown in Figure 1B. We found that some patients showed a significant improvement in albumin excretion, with their albuminuria resolving. In the patient who had macroalbuminuria at baseline, six months after the initiation of imeglimin, progressive DN ameliorated to the microalbuminuria level, suggesting a beneficial effect of imeglimin. We attributed the unmeasurable value of UACR to measurement sensitivity as the lower limit value, and UACR levels tended to decrease six months after treatment (P = 0.45).

Effect of imeglimin on NAFLD

**Figure 2:**
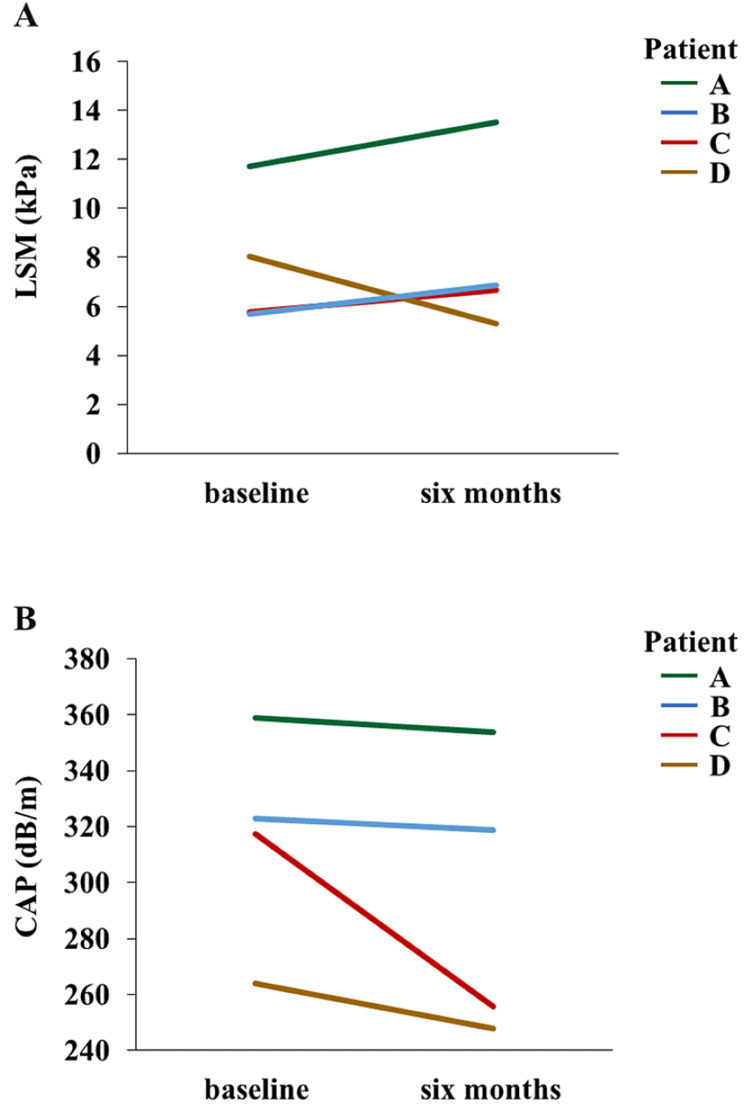
Changes in FibroScan data from baseline to six months after imeglimin treatment. A: The changes of LSM from baseline to six months after imeglimin treatment. B: The changes of CAP from baseline to six months after imeglimin treatment. P values are calculated by a paired t-test (vs. baseline parameters). LSM, liver stiffness measurement; CAP, controlled attenuation parameter

Four patients had NAFLD at baseline and underwent a FibroScan examination (Figure 2). The average LSM and CAP were 7.80 kPa and 316 dB/m, respectively. Six months after the initiation of imeglimin, a follow-up FibroScan examination for LSM and CAP was conducted. LSM was 8.10 kPa (P = 0.79), and CAP was 294 dB/m (P = 0.21).

Side effect of imeglimin

None of the patients discontinued imeglimin treatment during the study period. Some of the patients complained of self-limiting nausea or fatigue. Laboratory data showed no new evidence of side effects of imeglimin. None of the patients complained of hypoglycemia, suggesting the potential safety of this drug in preventing hypoglycemia.

## Discussion

The global prevalence of DM in 2015 was estimated at 415 million people, reaching 642 million by 2040 [18]. DM is not only a healthcare problem but also a social and economic burden; the American Diabetes Association estimated that its annual cost was 327 billion dollars in 2017 [19] and is still rising. In Japan, one in 10 adults is estimated to suffer from DM [20], and this condition has a great impact on the continuance of universal health insurance, which is a unique and valuable system based on the mutual aid of the people. Theoretically, lifestyle modification should be the first line of action for the management of DM. However, the widespread adoption of the Western lifestyle has hindered positive and healthy lifestyle changes. Many diabetes prevention trials have been conducted, and some have achieved successful results [21]; nevertheless, we have not been able to eradicate this disease, and it continues to spread explosively and globally.

Serious manifestations of DM include vascular complications. Hyperglycemia and hyperinsulinemia induce alterations in vascular tissues at the cellular level, leading to atherosclerosis [22]. Atherosclerotic changes lead to the disruption of blood flow or fragility of vessels; consequently, fatal diseases such as myocardial infarction or cerebral apoplexy can occur. Other microvascular complications such as retinopathy and neuropathy also restrict patients’ well-being and impair their quality of life.

Renal failure due to DN and eventual ESRD are other complications because most patients are forced to undergo hemodialysis, which incurs a great deal of medical expenses. DN is thought to occur due to metabolic and hemodynamic abnormalities [4]. Patients with DN initially show high GFR values, namely, hyperfiltration and microalbuminuria. Glomerular cells and podocytes are injured by the inflammatory cytokines TGFβ and disturbance of tubuloglomerular feedback provoked by hyperglycemia and glomerular hypertension [23]. Other factors such as advanced glycation end products and mechanical stretching have also been suggested to be involved in this process [24]. While the development of DN takes approximately 10-20 years from the onset of DM, initial microalbuminuria could enter remission. Once DN progresses to an advanced stage, further recovery is rendered impossible. In the present study, some patients had advanced proteinuria. After initiation of imeglimin treatment, proteinuria tended to decrease, and the DN stage improved to the former level. It is believed that the recovery of renal function takes a certain time period; however, in this study, the condition of DN improved in a relatively short time. This may be due to the hidden pharmacological effects of imeglimin beyond glycemic control, such as the obliteration of reactive oxygen species (ROS) or another specific mechanism. This was an observational study, and the true factor of improvement in DN remains to be understood; however, the effectiveness of imeglimin in DN management is strongly implied.

In patients with DM, NAFLD is a common comorbidity, and the prevalence is estimated to be approximately 30% [25]. Although the etiology of NAFLD has not been completely elucidated, insulin resistance and subsequent hyperinsulinemia are believed to play pivotal roles in its development. Insulin resistance is an exact definition of T2DM, and once T2DM worsens, the condition of NAFLD also deteriorates and vice versa. NAFLD itself is considered a risk factor for cardiovascular diseases; therefore, the prevention of its deterioration is an urgent concern. In accordance with the evidence of the histological improvement, NAFLD with DM is often treated with pioglitazone, a peroxisome proliferator-activated receptor γ agonist. Pioglitazone causes adipocytes to produce adiponectin, leading to reduced insulin resistance [26]. In addition, a positive effect of ipragliflozin on the pathology of NAFLD was recently demonstrated [27]. Although the effectiveness of these drugs for NAFLD coexisting with DM has been recognized, their use has not yet been approved owing to the difficulty in diagnosis. At present, the gold standard for the assessment of NAFLD is a liver biopsy; however, it is not easy to apply this test to all patients with NAFLD because of its high invasiveness. Several scoring systems for NAFLD have been proposed. As such, FLI has been developed as an easy assessment method for NAFLD screening [16] and can be calculated using general test values and patients’ demographic information. Despite their ease of use, the specificity of these scoring systems for NAFLD diagnosis remains low. Transient elastography is a noninvasive examination method used to evaluate liver stiffness and assess hepatic fibrosis [17]. This method can be easily performed during outpatient visits, and the data are immediately available. There is a solid relationship between biopsy results and elastography data [28]; therefore, we assessed the elastography data to evaluate the NAFLD condition. Elastography can also estimate the amount of fat in the liver as a CAP score; therefore, we used it to evaluate the NAFLD state of the patients. In this study, the treatment period was very short and showed no improvement in liver stiffness. However, imeglimin was shown to improve fat content in patients with NAFLD. Continuous imeglimin usage over a long treatment period would reveal the amelioration of liver impairment and stiffness. This drug may have beneficial effects on NAFLD and may be a potential therapeutic candidate.

Imeglimin is a novel glucose-lowering agent whose mechanism has not been fully elucidated; however, its main target is thought to be the mitochondria [11]. Mitochondria are essential organelles that function as powerhouses of ATP production via cellular respiration. A large amount of oxygen is utilized in ATP synthesis in the mitochondria, and reactive oxygen species (ROS) are generated as by-products of this process. Excessive ROS causes irreversible damage to DNA, proteins, and lipids, leading to the disruption of normal cellular functions [29]. In T2DM, ROS production is augmented by hyperglycemia, and various organs and cells are damaged or apoptosed [30]. In the pancreas, β cell dysfunction is provoked by ROS, and in the liver and muscles, the insulin signal deteriorates, leading to increased insulin resistance. Hence, the recovery of mitochondrial function is the most reasonable treatment for T2DM.

In this study, imeglimin was found to be a promising drug for the treatment of T2DM. The improvement in HbA1c levels in the participants was drastic during only six months. In the TIMES study [12-14], the effect on HbA1c levels was milder in the monotherapy group than in the combination therapy group. In our study, two patients received imeglimin monotherapy, but the efficacy was not inferior to that of the combination therapy.

It also has promising effects on body weight control. Interestingly, imeglimin improved DN and NAFLD. Considering the short-term improvement in these conditions, the end-organ protective effect of imeglimin may occur due to factors other than glycemic control. Pharmacological effects, such as the removal of reactive oxygen species or activation of the NAMPT gene, may be involved in these outcomes.

This study had several limitations. First, this was a single-center retrospective observational study, and we did not have a control group. Hence, there may be factors confounding the results, and the results may not be applicable to the general population. However, further studies are required to confirm the effects of imeglimin. Mild side effects were not confirmed and may have been overlooked. Second, the sample size was small, and the follow-up period was short (six months), which may have resulted in an inadequate observational period. Third, nine out of 10 participants were men, causing the gender ratio to be disproportionate. Therefore, a case analysis with an appropriate gender ratio is desired.

## Conclusions

In this study, we observed the efficacy of imeglimin for the treatment of T2DM. In addition to glycemic control, imeglimin can potentially improve DN and NAFLD. This drug could be a promising medication for patients with T2DM with comorbid DN and NAFLD. However, the small sample size and the short study period might have difficulty in generalizing the results. Therefore, further studies are needed to assess the effectiveness of this drug on glycemic control, as well as potential beneficial effects on DN and NAFLD, and the long-term efficacy of imeglimin on other vascular complications.

## References

[1] Nathan DM, DCCT/EDIC Research Group (2014). The diabetes control and complications trial/epidemiology of diabetes interventions and complications study at 30 years: overview. Diabetes Care.

[2] Stratton IM, Adler AI, Neil HA (2000). Association of glycaemia with macrovascular and microvascular complications of type 2 diabetes (UKPDS 35): prospective observational study. BMJ.

[3] Roglic G (2016). WHO global report on diabetes: a summary. Int J Noncommun Dis.

[4] Dronavalli S, Duka I, Bakris GL (2008). The pathogenesis of diabetic nephropathy. Nat Clin Pract Endocrinol Metab.

[5] Lewis EJ, Hunsicker LG, Bain RP, Rohde RD (1993). The effect of angiotensin-converting-enzyme inhibition on diabetic nephropathy. The collaborative study group. N Engl J Med.

[6] Ruggenenti P, Cravedi P, Remuzzi G (2010). The RAAS in the pathogenesis and treatment of diabetic nephropathy. Nat Rev Nephrol.

[7] Neuen BL, Young T, Heerspink HJL (2019). SGLT2 inhibitors for the prevention of kidney failure in patients with type 2 diabetes: a systematic review and meta-analysis. Lancet Diabetes Endocrinol.

[8] Bakris GL, Agarwal R, Anker SD (2020). Effect of finerenone on chronic kidney disease outcomes in type 2 diabetes. N Engl J Med.

[9] Friedman SL, Neuschwander-Tetri BA, Rinella M, Sanyal AJ (2018). Mechanisms of NAFLD development and therapeutic strategies. Nat Med.

[10] Targher G, Byrne CD, Tilg H (2020). NAFLD and increased risk of cardiovascular disease: clinical associations, pathophysiological mechanisms and pharmacological implications. Gut.

[11] Vial G, Chauvin MA, Bendridi N (2015). Imeglimin normalizes glucose tolerance and insulin sensitivity and improves mitochondrial function in liver of a high-fat, high-sucrose diet mice model. Diabetes.

[12] Dubourg J, Fouqueray P, Thang C, Grouin JM, Ueki K (2021). Efficacy and safety of imeglimin monotherapy versus placebo in Japanese patients with type 2 diabetes (times 1): a double-blind, randomized, placebo-controlled, parallel-group, multicenter phase 3 trial. Diabetes Care.

[13] Dubourg J, Fouqueray P, Quinslot D, Grouin JM, Kaku K (2022). Long-term safety and efficacy of imeglimin as monotherapy or in combination with existing antidiabetic agents in Japanese patients with type 2 diabetes (times 2): a 52-week, open-label, multicentre phase 3 trial. Diabetes Obes Metab.

[14] Reilhac C, Dubourg J, Thang C, Grouin JM, Fouqueray P, Watada H (2022). Efficacy and safety of imeglimin add-on to insulin monotherapy in Japanese patients with type 2 diabetes (times 3): a randomized, double-blind, placebo-controlled phase 3 trial with a 36-week open-label extension period. Diabetes Obes Metab.

[15] Lamb YN (2021). Imeglimin hydrochloride: first approval. Drugs.

[16] Vallet-Pichard A, Mallet V, Nalpas B (2007). FIB-4: an inexpensive and accurate marker of fibrosis in HCV infection. Comparison with liver biopsy and fibrotest. Hepatology.

[17] Sandrin L, Fourquet B, Hasquenoph JM (2003). Transient elastography: a new noninvasive method for assessment of hepatic fibrosis. Ultrasound Med Biol.

[18] Ogurtsova K, da Rocha Fernandes JD, Huang Y (2017). IDF diabetes atlas: global estimates for the prevalence of diabetes for 2015 and 2040. Diabetes Res Clin Pract.

[19] American Diabetes Association (2018). Economic costs of diabetes in the U.S. in 2017. Diabetes Care.

[20] (2024). Japan diabetes report 2000-2045. https://www.diabetesatlas.org/data/en/country/101/jp.html.

[21] American Diabetes Association Professional Practice Committee (2024). 3. Prevention or delay of diabetes and associated comorbidities: standards of care in diabetes-2024. Diabetes Care.

[22] Rask-Madsen C, King GL (2013). Vascular complications of diabetes: mechanisms of injury and protective factors. Cell Metab.

[23] Yamamoto T, Nakamura T, Noble NA, Ruoslahti E, Border WA (1993). Expression of transforming growth factor beta is elevated in human and experimental diabetic nephropathy. Proc Natl Acad Sci U S A.

[24] Fukami K, Yamagishi S, Ueda S, Okuda S (2008). Role of AGEs in diabetic nephropathy. Curr Pharm Des.

[25] Chalasani N, Wilson L, Kleiner DE, Cummings OW, Brunt EM, Unalp A, NASH Clinical Research Network (2008). Relationship of steatosis grade and zonal location to histological features of steatohepatitis in adult patients with non-alcoholic fatty liver disease. J Hepatol.

[26] Sanyal AJ, Chalasani N, Kowdley KV (2010). Pioglitazone, vitamin E, or placebo for nonalcoholic steatohepatitis. N Engl J Med.

[27] Takahashi H, Kessoku T, Kawanaka M (2022). Ipragliflozin improves the hepatic outcomes of patients with diabetes with NAFLD. Hepatol Commun.

[28] Yoneda M, Yoneda M, Fujita K, Inamori M, Tamano M, Hiriishi H, Nakajima A (2007). Transient elastography in patients with non-alcoholic fatty liver disease (NAFLD). Gut.

[29] Srinivas US, Tan BWQ, Vellayappan BA, Jeyasekharan AD (2019). ROS and the DNA damage response in cancer. Redox Biol.

[30] Volpe CMO, Villar-Delfino PH, Dos Anjos PMF, Nogueira-Machado JA (2018). Cellular death, reactive oxygen species (ROS) and diabetic complications. Cell Death Dis.

